# Gut Microbiota Eubacterium callanderi Exerts Anti-Colorectal Cancer Activity

**DOI:** 10.1128/spectrum.02531-22

**Published:** 2022-11-30

**Authors:** Seoung Woo Ryu, Ji-Sun Kim, Byeong Seob Oh, Won Jung Choi, Seung Yeob Yu, Jeong Eun Bak, Seung-Hwan Park, Se Won Kang, Jiyoung Lee, Won Yong Jung, Jung-Sook Lee, Ju Huck Lee

**Affiliations:** a Korean Collection for Type Cultures, Biological Resource Center, Korea Research Institute of Bioscience and Biotechnology, Jeongeup, Republic of Korea; b Korean Bioinformation Center, Korea Research Institute of Bioscience and Biotechnology, Daejeon, Republic of Korea; Nanchang University

**Keywords:** gut microbiota, *Eubacterium callanderi*, colorectal cancer, anti-cancer activity, apoptosis, cell cycle arrest

## Abstract

The gut microbiota (GM) is associated with colorectal cancer (CRC) development. However, studies demonstrating the role of GM in CRC are limited to metagenomic analyses. These studies lack direct evidence proving that the candidate strains are involved in CRC, and isolated probiotics for bacteriotherapy. Therefore, to identify novel GM with anti-CRC activity, we previously isolated gut bacteria from the feces of healthy individuals, screened the isolated GM’s anti-CRC activity, and discovered that cell-free supernatants of GM isolates demonstrated antiproliferative activity against CRC cells. Here, our study identified one of them as Eubacterium callanderi and chose it for further study because the genus *Eubacterium* has been suggested to contribute to various aspects of gut health; however, the functions are unknown. First, we confirmed that *E. callanderi* cell-free supernatant (EcCFS) exerted antiproliferative activity—by inducing apoptosis and cell cycle arrest—that was dose-dependent and specific to cancer cell lines. Next, we discovered that EcCFS active molecules were heat stable and protease insensitive. High-performance liquid chromatography analysis revealed that EcCFS contained high butyrate concentrations possessing anticancer activity. Additionally, gas chromatography-mass spectrometry analysis of the aqueous phase of ethyl acetate-extracted EcCFS and an antiproliferation assay of the aqueous phase and 4-aminobutanoic acid (GABA) suggested that GABA is a possible anti-CRC agent. Finally, in the CT26 allograft mouse model, *E. callanderi* oral administration and EcCFS peri-tumoral injection inhibited tumor growth *in vivo*. Therefore, our study reveals that *E. callanderi* has an anti-CRC effect and suggests that it may be a potential candidate for developing probiotics to control CRC.

**IMPORTANCE** The gut microbiota has been reported to be involved in colorectal cancer, as suggested by metagenomic analysis. However, metagenomic analysis has limitations, such as bias in the analysis and the absence of bacterial resources for follow-up studies. Therefore, we attempted to discover gut microorganisms that are related to colorectal cancer using the culturomics method. In this study, we discovered that Eubacterium callanderi possesses anti-colorectal cancer activity *in vitro* and *in vivo*, suggesting that *E. callanderi* could be used in bacteriotherapy for colorectal cancer treatment.

## INTRODUCTION

The gut microbiota (GM) and host have symbiotic interactions that benefit and harm the host in many ways (1). While the balance of gut microorganisms is critical for maintaining host health, the shift of gut bacterial composition called dysbiosis is associated with various diseases based on metagenomic analysis. Clostridioides difficile infection (CDI), for instance, was revealed to lower the diversity of GM and reduce the abundance of *Roseburia*, *Blautia*, and *Lachnospiraceae* members in metagenomics study (2). Additionally, GM is related to host immunity and affects immune diseases, such as inflammatory bowel disease (IBD). A metagenomics-based study revealed that patients with IBD have reduced diversity of GM and depleted butyrate-producing bacteria compared with the diversity and number in healthy controls (3). Furthermore, gut microorganisms can influence metabolic diseases such as obesity. Obesity groups had a decreased *Bacteroidetes* population, reducing bacterial diversity (4). These data provide evidence that GM is related to host fitness. Metagenomics has revolutionized gut microbiome research, and sequencing technology has advanced. However, 16S rRNA gene amplicon-based metagenomic studies contain biases that are generated during the sequencing and bioinformatic processes such as DNA extraction, primer region selection, cell viability, and program methodology, making the determination of the involvement of GMs in human health a challenge (5). Another drawback of metagenomics is that it cannot provide bacteria, which could be potential candidates for bacteriotherapy as probiotics, for further studies (5). In contrast, culturomics based on isolating GM can overcome the limitations of metagenomic analysis and secure the actual bacterial strains for further research. Therefore, we used culturomics that involves isolation of gut bacterial strains from healthy individuals to verify or determine the function of GM in host fitness.

Colorectal cancer (CRC) is related to GM, and gut dysbiosis contributes to CRC development (6, 7). Based on metagenomic analyses, many studies have revealed that *Eubacterium* and *Roseburia* spp. levels are significantly lower in patients with CRC than in healthy individuals (8 to 10). Furthermore, the involvement of GM in CRC was verified using isolated bacteria, such as *Lactobacillus* spp. and Akkermansia muciniphila (11 to 13). In addition, we recently demonstrated that Odoribacter splanchnicus exhibits anti-CRC activity (14). However, the metagenomic analysis of patients with CRC can have the same limitations mentioned above; thus, culture-based identification is needed to identify gut microbes for CRC treatment.

*Eubacterium* spp. are Gram-positive, rod-shaped, non-spore-forming, obligate anaerobes mainly isolated from the oral cavity and intestinal tract (15). Members of the genus *Eubacterium* form part of the core human gut microbiome and have been recognized as potentially beneficial microbes (16). There are 26 validly published species in *Eubacterium*, but the core genotypes of this genus are restricted to Eubacterium limosum, Eubacterium callanderi, Eubacterium barkeri, and Eubacterium aggregans (17, 18). *Eubacterium* spp. have been linked to human health, including the development of CRC. With regard to CRC development, E. rectale, E. hallii, and E. ventriosum are significantly reduced in patients with CRC (8, 19), and metagenomic studies have revealed that *E. ventriosum* is consistently enriched in control groups, suggesting that it can be used as a biomarker for low CRC risk (20). Furthermore, *Eubacterium* spp. are short-chain fatty acid (SCFA) producers, which produce butyrate, acetate, and formic acids as major SCFAs (15). Butyrate is known to inhibit carcinogenesis and induce cancer cell apoptosis (21). *E. callanderi* is a species of the genus *Eubacterium* that has been related to human health. However, there is no evidence showing the involvement of *E. callanderi* in human health. Therefore, it is necessary to explore the role of *E. callanderi* in host fitness.

Culture-based analysis was employed to determine the composition of the gut microbes. However, with advances in sequencing technology, metagenomics has been widely used to identify GM, particularly for comprehensive analysis of gut microbial populations. Interestingly, culturomics has recently been reemphasized for microbial identification owing to several drawbacks of metagenomic analysis, the most important of which is the lack of isolated microbes for further study (5). In this study, we isolated *E. callanderi* KGMB02377 from healthy Korean feces and investigated its role in host physiology. Our studies discovered the novel GM possessing anti-CRC activity, which could be a promising novel therapeutic probiotic for CRC treatment.

## RESULTS

### *E. callanderi* cell-free supernatant selectively inhibits tumor growth *in vitro*.

**FIG 1:**
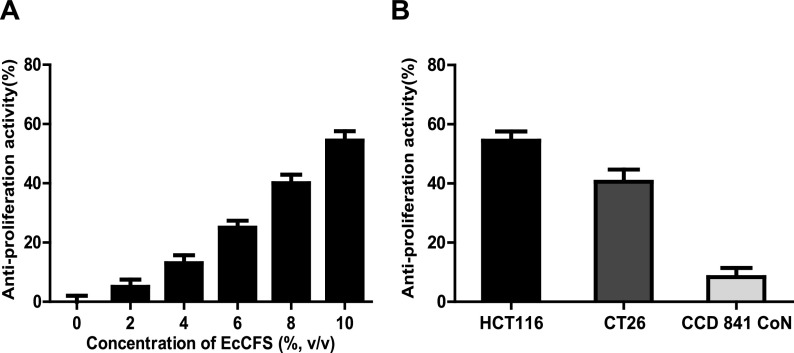
Selective antiproliferative effects of Eubacterium callanderi cell-free supernatant (EcCFS) in a cell-based system. (A) Dose-dependent antiproliferative effect of EcCFS on HCT116 cells. (B) Antiproliferative effect of EcCFS on cancer cell lines (HCT116 and CT26) and normal colon cell line (CCD 841 CoN). Each cell line was treated with 10% EcCFS. RCM broth was used as a control for antiproliferative activity calculation. Data are expressed as mean ± standard deviation of three independent experiments.

In a previous study, we screened the anti-CRC activity of gut microbes isolated from healthy Korean feces using their cell-free supernatants (CFSs) and discovered several CFSs with antiproliferative activity against colonic carcinoma cell lines (14). To identify one of the candidate microbes, we performed 16S rRNA gene sequencing and phylogenetic and phenotypic analyses, and discovered that it is *E. callanderi* (99.52% 16S rRNA gene similarity, rod-shaped without flagella). The phylogenetic tree revealed that isolated *E. callanderi* KGMB02377 is related to the type strain *E. callanderi* DSM 3662^T^, and this node was grouped with *E. limosum*, which is the type species of the genus *Eubacterium* (Fig. S1 in the supplemental material). *Eubacterium* has been involved in various human health-related diseases; hence, we examined whether EcCFS possessed inhibitory activities against several diseases such as CRC, IBD, CDI, and obesity (Fig. S2). EcCFS had activity against only the CRC cell line (Fig. S2A). Thus, we focused on the anti-CRC activity of *E. callanderi*. To confirm its antiproliferative activity, cells were treated with different doses of EcCFS (2% intervals from 0% to 10%). The results showed that the inhibition of human colon cancer cell line (HCT116) proliferation positively correlated with the concentration of EcCFS (Fig. 1A). Furthermore, to examine whether this activity was specific to cancer cells, a mouse colon cancer cell line (CT26) and normal colon epithelial cells (CCD 841 CoN) were treated with EcCFS. Interestingly, EcCFS demonstrated antiproliferative activity of 54.4% and 40.5% in HCT116 and CT26, respectively, but less than 10% growth inhibition was observed in CCD 841 CoN (Fig. 1B). Therefore, we concluded that EcCFS has selective inhibitory activity against the proliferation of CRC cells.

### EcCFS induces apoptosis and cell cycle arrest of the HCT116.

**FIG 2:**
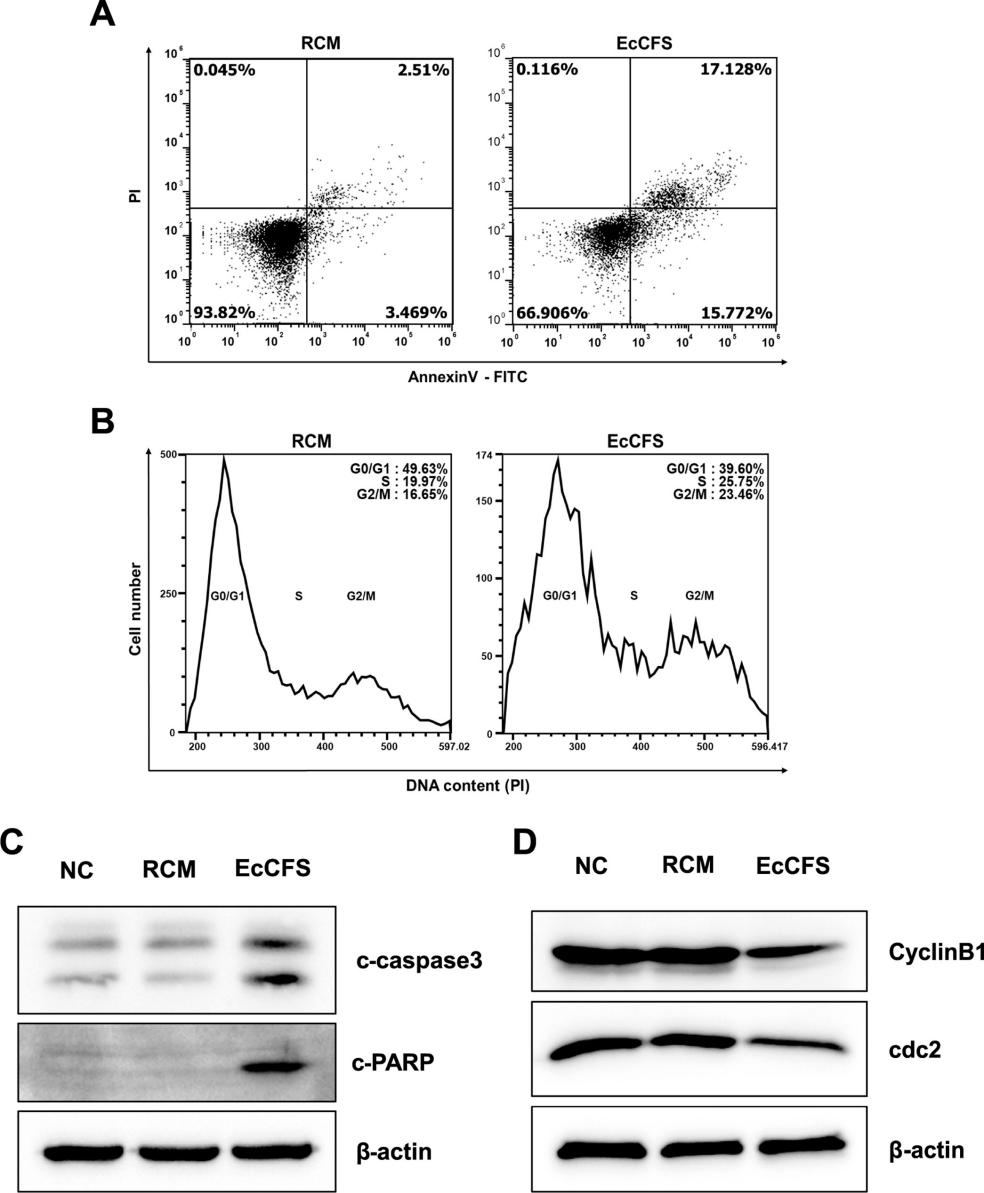
Flow cytometry and Western blot analysis of HCT116 cells treated with EcCFS. (A) Representative dot plots of apoptosis analysis using Annexin V-FITC and PI staining. (B) Representative histograms of cell cycle distribution using PI staining. (C) Western blot analysis of apoptosis marker proteins, cleavage forms of caspase3 (c-caspase3), and PARP (c-PARP). (D) Western blot analysis for G2/M cell cycle check point marker proteins cyclinB1 and cdc2. β-actin was used as loading control. RCM treatments were used as a control.

The general mechanisms of cell growth inhibition include apoptosis and cell cycle arrest (22, 23); therefore, these two pathways were investigated using flow cytometry and Western blot analysis to determine the molecular mechanism of the antiproliferative activity of EcCFS. To determine whether EcCFS activity was related to apoptosis, we measured apoptotic cell populations in EcCFS-treated HCT116 cells using FITC-Annexin V and propidium iodide (PI) double staining. The early- and late-stage apoptotic cell populations of EcCFS-treated HCT116 cells increased by 12.3% and 14.6%, respectively, compared to the populations of reinforced clostridial medium (RCM)-treated cells (Fig. 2A). Furthermore, to confirm the apoptotic activity of EcCFS, cleaved caspase3, a key regulator of apoptosis, and the cleaved form of its downstream target poly (ADP-ribose) polymerase (PARP) were examined by Western blot analysis. The analysis revealed that the levels of both cleaved proteins increased in EcCFS-treated cells compared with those in the control cells (Fig. 2C). The distribution of DNA contents in HCT116 cells treated with EcCFS or RCM was analyzed using flow cytometry to evaluate whether the antiproliferative activity of EcCFS is related to cell cycle arrest. Our results revealed that EcCFS-treated cells in the G2/M phase increased by approximately 6.8% compared with the percentage of RCM-treated cells in G2/M phase (Fig. 2B). Similarly, Western blot analysis verified the cell cycle arrest activity of EcCFS, revealing that the expression of cdc2 and cyclinB1, the G2/M checkpoints, decreased in EcCFS-treated cells compared with that in the RCM-treated cells (Fig. 2D). These results suggest that EcCFS exhibits antiproliferative activity through apoptosis and G2/M phase cell cycle arrest.

### Metabolites butyrate and putative GABA may be the active molecules of EcCFS.

**FIG 3:**
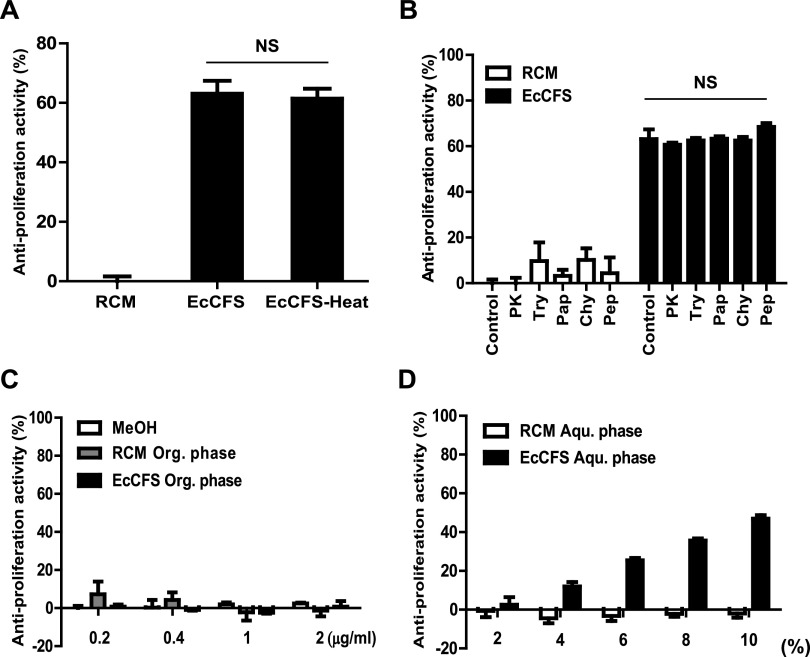
Characterization of the active molecules of EcCFS. (A and B) Effects of heat and protease treatments on antiproliferative activity of EcCFS. (C and D) Effects of EtOAc extraction on antiproliferative activity of EcCFS. The organic phase (Org. phase) of the extract was dissolved in MeOH, which was used as a control, and was administered with increasing dose (C). Cells were treated with different doses of the aqueous phase (Aqu. phase) of EcCFS (2 to 10%, 2% interval) (D). PK, proteinase K; Try, trypsin; Pap, papain; Chy, chymotrypsin; Pep, pepsin. Data are expressed as mean ± standard deviation of three independent experiments.

Bacterial CFS contains many active molecules, such as metabolites and proteins (24). We examined the features of the components to identify the active agents responsible for the antiproliferative activity of EcCFS. First, we boiled EcCFS to check for heat sensitivity of the active molecules. However, the antiproliferative activity of EcCFS was not influenced by heat treatment (Fig. 3A). Next, we treated EcCFS with various proteases to determine whether the active agents were large protein molecules. Since cytotoxicity was observed by the treatments of some proteases to the cells and EcCFS activity was not affected by heat treatment, the protease-treated EcCFSs were boiled before antiproliferative activity assay. As shown in Fig. 3B, none of the proteases affected the antiproliferative activity of EcCFS (Fig. 3B). Finally, we investigated whether organic solvents could extract the active antiproliferative molecules of EcCFS. Ethyl acetate (EtOAc) was used to extract bioactive molecules from EcCFS. The organic phase of the EtOAc extract did not show antiproliferative activity; however, the aqueous phase of the extract exhibited this activity in a dose-dependent manner (Fig. 3C and D). Based on these analyses, we assumed that the agents with antiproliferative activity might be metabolites possessing heat and protease stability properties.

**FIG 4:**
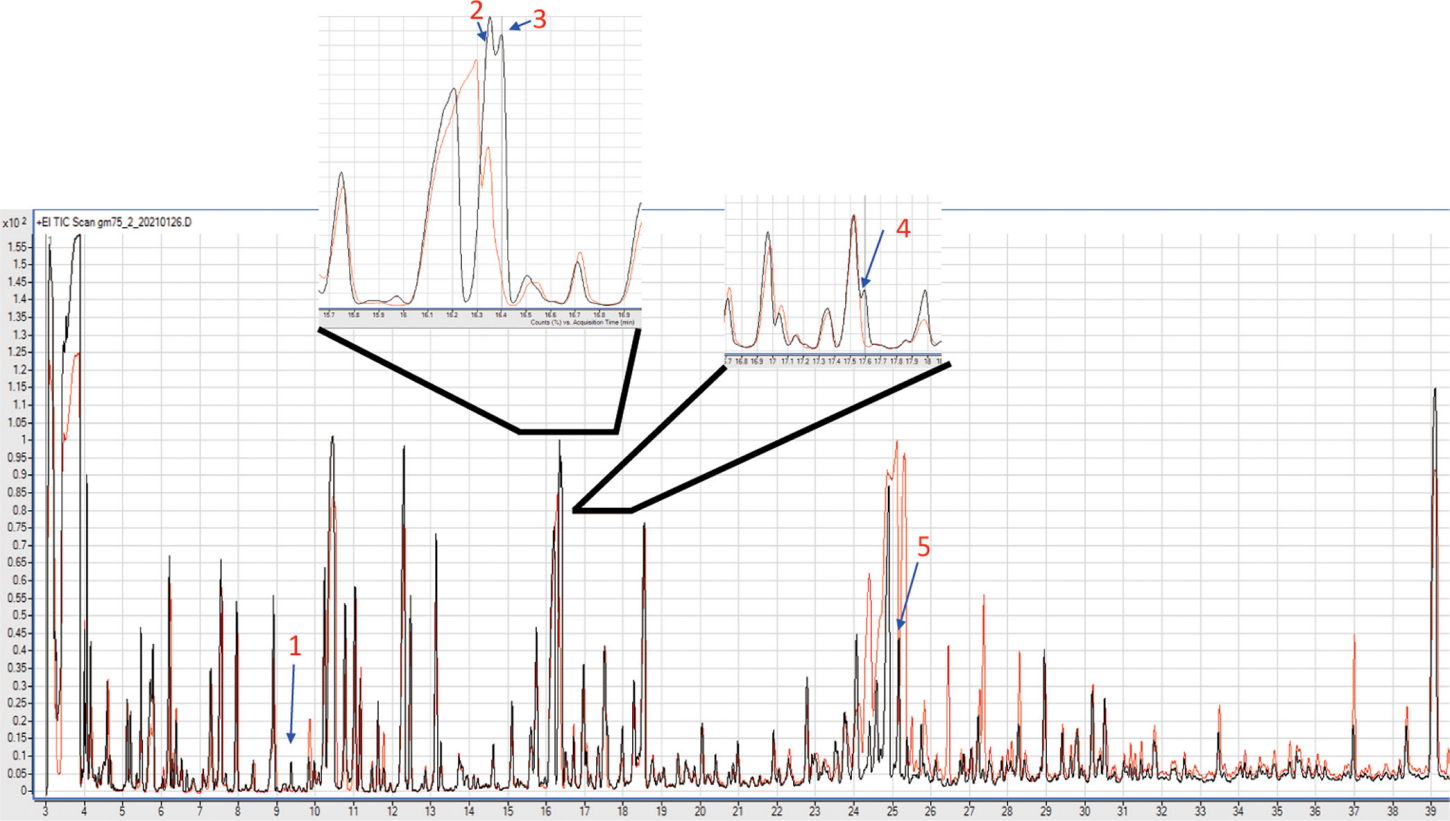
Comparison of GC-MS chromatogram between the aqueous phase of RCM and EcCFS after EtOAc extraction. The different peaks with *P* value of <0.005 from the comparison of GC-MS chromatogram were selected (nos. 1 to 5); the peaks for 2, 3, and 4 are magnified for a clearer view. Red and black graphs indicate RCM as a control and EcCFS, respectively.

*Eubacterium* spp. are butyrate producers, and butyrate is known to inhibit cancer cell growth; the antiproliferative assay of butyrate showed that it could successfully inhibit the proliferation of the CRC cells in a dose-dependent manner (Fig. S3A) and is a protease-stable metabolite. Therefore, we evaluated whether EcCFS contained butyrate. High-pressure liquid chromatography (HPLC) analysis of SCFAs in the EcCFS revealed that a large amount of butyrate was present, while other SCFAs were not detected, except a small amount of acetic acid (Table 1). Interestingly, 1 mM butyrate treatment showed a similar activity compared with EcCFS treatment (10% EcCFS in Dulbecco’s modified Eagle’s medium [DMEM]), suggesting that butyrate may be a key active molecule for the anti-CRC activity of EcCFS. Additionally, we employed gas chromatography-mass spectrometry (GC-MS) analysis of the aqueous phase of the EtOAc extract to investigate more candidates as antiproliferative active agents of EcCFS. The peak difference of the aqueous phase of EcCFS compared with that of RCM as a control was analyzed by GC, and five distinct peaks were revealed and identified by MS (Fig. 4). According to the National Institute of Standards and Technology library, peak 1 (t_R_ 9.38 min), peak 2 (t_R_ 16.35 min), peak 3 (t_R_ 16.4 min), peak 4 (t_R_ 17.6 min), and peak 5 (t_R_ 25.17 min) were identified as l-hydroxyisocaproic acid, l-aspartic acid, 4-aminobutanoic acid, 3-phenyllactic acid, and l-tyrosine, respectively (Table 2). Among the candidates from GC-MS analysis, 4-aminobutanoic acid, also known as γ-aminobutyric acid (GABA), had the highest probability, suggesting that the antiproliferative agent of EcCFS might be GABA. Interestingly, the antiproliferative assay of GABA revealed that it could successfully inhibit the proliferation of the CRC cells in a dose-dependent manner (Fig. S3B).

**TABLE 1:** Concentration of SCFAs in *E. callanderi* cell-free supernatant (EcCFS)^*a*^

Short-chain fatty acids (SCFAs)	Concn of SCFAs (mM)
Lactic acid (LA)	-
Acetic acid (AA)	0.14
Propionic acid (PA)	-
Butyric acid (BA)	10.20

a Concn, concentration; -, below the quantification value.

**TABLE 2:** Candidate antiproliferative active molecules of EcCFS based on GC-MS analysis

No.	Time	Match	R match	Prob (%)	Identification	Formula	*P* value
1	9.38	923	957	73.9	L-Hydroxyisocaproic acid, 2TMS derivative	C_12_H_28_O_3_SI_2_	0.0008
2	16.35	746	493	71.1	L-Aspartic acid, 2TMS derivative	C_13_H_31_NO_4_SI_3_	0.0005
3	16.4	930	930	94.9	4-Aminobutanoic acid, 2TMS derivative	C_13_H_33_NO_2_SI_3_	0.0002
4	17.6	898	954	81.0	3-Phenyllactic acid, 2TMS derivative	C_15_H_26_O_3_SI_2_	<0.0001
5	25.17	907	925	90.9	L-Tyrosine, 2TMS derivative	C_18_H_35_NO_3_SI_3_	<0.0001

**FIG 5:**
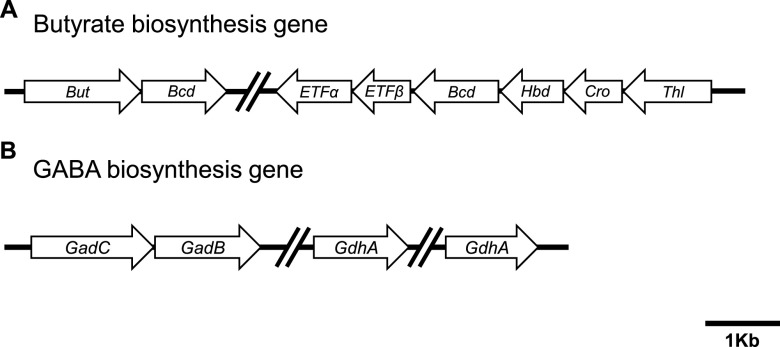
Butyrate and GABA biosynthesis genes based on whole-genome sequencing analysis of *E. callanderi* KGMB02377. (A) Series of genes related to butyric acid biosynthesis. *But*, butyryl-CoA:acetate CoA transferase (locus_tag, KR505_02670); *Bcd*, butyryl-CoA dehydrogenase (KR505_02675, KR505_10030); *ETFα*, electron transfer flavoprotein subunit alpha (KR505_10020); *ETFβ*, electron transfer flavoprotein subunit beta (KR505_10025); *Hbd*, hydroxybutyryl dehydrogenase (KR505_10035); *Cro*, crotonase/enoyl-CoA hydratase (KR505_10040); *Thl*, acetyl-CoA C-acetyltransferase (KR505_10045). (B) Series of genes related to GABA biosynthesis. *GadC*, glutamate/gamma-aminobutyrate antiporter (KR505_12475); *GadB*, glutamate decarboxylase (KR505_12480); *GdhA*, glutamate dehydrogenase (NADP^+^) (KR505_13925, KR505_18950). Scale bar: 1 Kb.

The biochemical analyses for the identification of the active molecules of EcCFS suggested that the putative agents of EcCFS exerting antiproliferative activity are butyrate and GABA; hence, we conducted genomic analysis to investigate whether the genome of the isolated *E. callanderi* KGMB02377 contains genes for butyrate and GABA synthesis. Here, the whole-genome sequencing (WGS) of KGMB02377 was performed. WGS revealed that the length of the genome was 4,673,496 bp, the DNA G+C content was 47.2 mol%, and the genome contained 4,429 open reading frames, five rRNA genes, and 43 tRNA genes (Table S1). The genome was 5% longer in length (224,603 bp) but had a similar G+C content compared to the genome of the type strain *E. callanderi* FD^T^ (= DSM 3662^T^). Moreover, the genome had 18 clusters of orthologous groups of proteins, among which those with unknown function accounted for the largest gene number (35.0%), followed by those related to transcription (9.6%), amino acid transport and metabolism (7.9%), and energy production and conversion (6.4%) (Table S2). Interestingly, we discovered that KGMB02377 contains a series of genes for the synthesis of butyrate from acetyl-CoA (Fig. 5A). Additionally, we searched for genes involved in GABA bioconversion. GABA is synthesized from glutamate by glutamate decarboxylase (*gadA*/*gadB*), and glutamate is synthesized from α-ketoglutarate by glutamate dehydrogenase (*gdhA*). The *gadC* gene encodes a glutamate/GABA antiporter (25). We identified *gadB*, putative *gadC*, and two *gdhA* genes in the genome of KGMB02377 (Fig. 5B). Therefore, our biochemical and genomic analyses indicated that butyrate and GABA are putative bioactive molecules of EcCFS with antiproliferative activity.

### *E. callanderi* live bacteria and EcCFS inhibit tumor progression *in vivo*.

**FIG 6:**
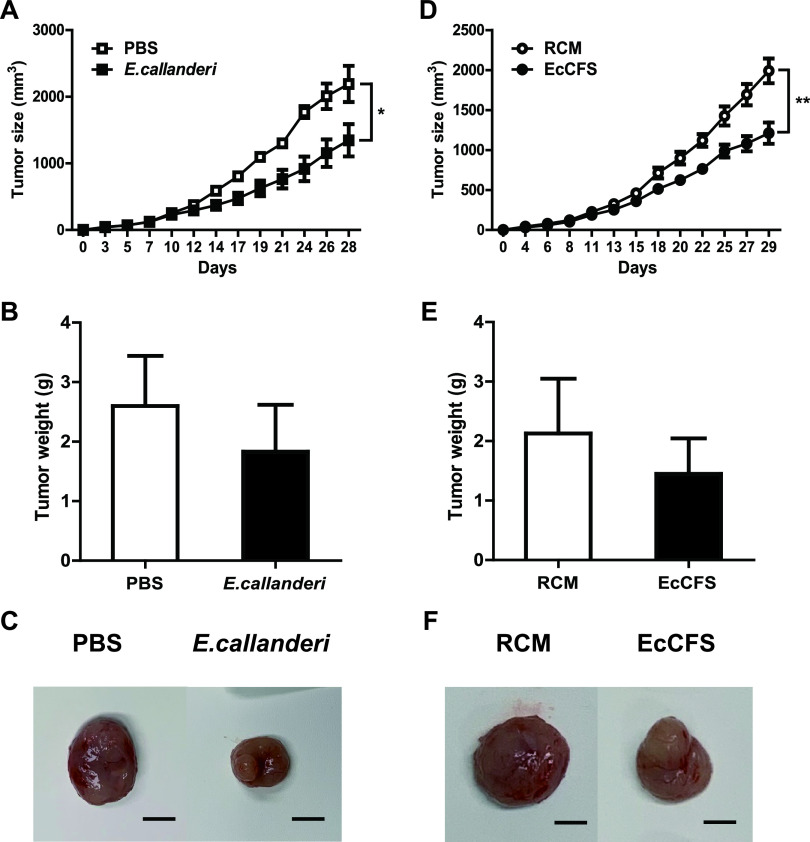
*In vivo* effects of *E. callanderi* in colorectal cancer mouse model. Left and right figures represent the oral administration and peri-tumoral injection, respectively. PBS and RCM were used as control for live *E. callanderi* (oral administration) and EcCFS (peri-tumoral injection), respectively. (A and D) Graphs of the tumor volume of mice for the oral administration (A) and peri-tumoral injection (D). (B and E) Tumor weights of mice at necropsy for the oral administration (B) and peri-tumoral injection (E). (C and F) The representative photographs of excised tumors at necropsy for the oral administration (C) and peri-tumoral injection (F). Scale bars: 1 cm. Data are expressed as mean ± standard deviation of these groups (each group = 5 mice).

We used an established CT26 murine allograft model for oral treatment and peri-tumoral injection to investigate the anti-CRC activity of *E. callanderi in vivo* (Fig. S4). When *E. callanderi* live bacteria were orally administered, tumor growth was suppressed compared with that in the control group. At the end of the *in vivo* experiment, the tumor size of the bacteria-treated group reduced significantly by 38.6% and their tumor weight also decreased by 29.5%, compared to the values of the phosphate-buffered saline (PBS)-treated control group (Fig. 6A to C). Similarly, when EcCFS was administered to the peripheral region of the tumor, tumor proliferation suppressed by 39.2% and the weight decreased by 32% compared with the corresponding values in the RCM-treated control group. (Fig. 6D to F).

**FIG 7:**
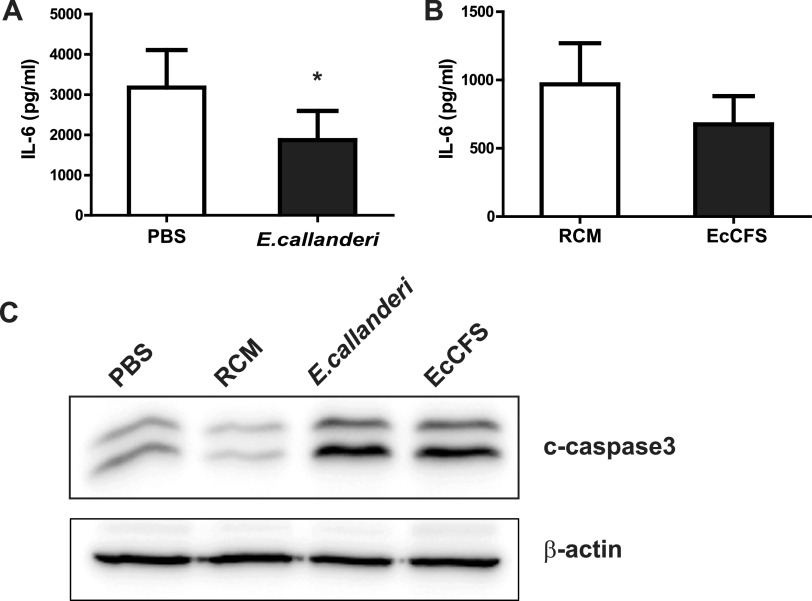
Effects of *E. callanderi* on mice inflammation and tumor apoptosis. (A and B) Serum IL-6 levels of the mice for the oral administration (A) and peri-tumoral injection (B). (C) The expression level of cleaved caspase3 in the tumor detected by Western blot analysis.

The interleukin-6 (IL-6) has been reported to be a crucial tumor-promoting cytokine, and its expression is significantly increased in colorectal carcinoma (26, 27). Therefore, IL-6 levels in mouse serum were measured using enzyme-linked immunosorbent assay (ELISA). IL-6 level in the serum of the live bacteria-administered group reduced significantly by 41.2%, and EcCFS-treated groups’ IL-6 level also decreased by 30.1%, compared to those in the control group (Fig. 7A and B). Additionally, when caspase3 was examined by Western blot analysis in tumor tissue, level of the cleaved form of caspase3 was found to have increased in the live bacteria and EcCFS-administered samples compared to that in the control samples (Fig. 7C). Thus, these results indicate that the administration of *E. callanderi* live bacteria and EcCFS exhibited anti-CRC activity *in vivo*, leading to the reduction of IL-6 levels in serum and tumor apoptosis.

## DISCUSSION

Recent metagenomic studies have revealed that GM is associated with CRC (28). Dysbiosis promotes the development and progression of CRC by altering the composition of GM (6). In patients with CRC, the genera *Bacteroides*, *Roseburia*, and *Eubacterium* were reduced compared to the corresponding levels in the healthy volunteers (8). Another study revealed that the control group had higher levels of the genera *Ruminococcus*, *Bifidobacterium*, and Streptococcus, which were depleted in the CRC group (29). However, it cannot be concluded that the above candidates selected by metagenomic analysis are helpful in preventing or curing CRC, because of the biases generated during the analysis and absence of isolated microbes for further study. Therefore, culturomic-based studies have recently been reemphasized to overcome the limitations of metagenomics, most importantly to obtain resources for bacteriotherapy. For example, in a culture-based study on CRC treatment, oral administration of Lactobacillus casei BL23 to CRC mice revealed antitumor effects that reduced histological scores and proliferative index values (11). Another study revealed that Lactobacillus delbrueckii CU/22 producing hydrogen peroxide could induce apoptosis and necrosis in human CRC cells (30). Moreover, our recent study demonstrated that *O. splanchnicus* exerts anti-CRC activity by inducing apoptosis (14). However, numerous anti-CRC candidates still need to be confirmed or identified. In this study, we identified a novel GM with anti-CRC activity *in vitro* and *in vivo*.

Based on a meta-analysis, genus *Eubacterium* was found to significantly decrease in many diseases (20, 31 to 33). Moreover, *Eubacterium* species are butyrate producers, which have many beneficial effects on host fitness. *E. callanderi* is a core *Eubacterium* species; however, its functions in host health have not yet been characterized. Therefore, we screened the effects of *E. callanderi* on human health. We used EcCFS to examine the inhibitory activity of this strain against CRC, IBD, CDI, and obesity in our established *in vitro* assays, because it has been reported that bacterial CFSs possess many bioactive agents influencing human health (34 to 37). From the screening data for the potential effect of *E. callanderi* on various diseases, only the antiproliferative effect on HCT116 could be confirmed (Fig. S2 and Fig. 1). However, functions other than antiproliferative activity cannot be ignored, because the activity of EcCFS may depend on the assay system, and other bacterial materials such as lysates and live or dead cells may influence the bioactivities of *E. callanderi*. Therefore, further studies are needed to elucidate whether *E. callanderi* possesses activities other than the anti-CRC activity discovered in this study.

We identified the mechanism underlying the antiproliferative activity of EcCFS. The proliferation of cell lines is affected by apoptosis and cell cycle arrest (38). Therefore, many cancer therapeutic drugs target the induction of apoptosis or cell cycle arrest (39, 40). In this study, we examined whether EcCFS stimulates apoptosis and cell cycle arrest using flow cytometry and Western blot analysis. Our data revealed that EcCFS inhibited the proliferation of HCT116 cells by inducing apoptotic cell death and G2/M phase cell cycle arrest (Fig. 2). Wan et al. reported that the fermentation supernatants of Lactobacillus delbrueckii induced G1 phase arrest and caspase3-dependent apoptosis. The supernatants not only inhibited the growth of colon cancer cells, but also antagonized invasion by reducing matrix-metalloproteinase-9 activity (41). Additionally, Lactobacillus plantarum 06CC2 extract activates c-Jun N-terminal kinase/p38 mitogen-activated protein kinase (JNK/p38 MAPK) signaling, leading to mitochondrion-mediated apoptosis in CRC cells (42). Another study revealed that the probiotic Bacillus polyfermenticus exerts anticancer activity by reducing ErbB2 and ErbB3 levels (43). The above reports suggest a mechanism of action for EcCFS; however, the exact mechanism needs to be further studied.

The active molecules of EcCFS with antiproliferative activity have not been completely identified; however, we discovered that they are heat stable and nonproteinaceous, suggesting that the active agents may be metabolites, not proteins (Fig. 3A and B). Thus, butyrate is a possible candidate because it is a metabolite, known to be an antiproliferative agent, our *in vitro* assay confirmed that butyrate showed antiproliferative activity (Fig. S3A), and *Eubacterium* spp. are butyrate-producers (44). In addition, since it has been reported that acidic pH is needed for efficient ethyl acetate extraction of butyrate (45), we checked the butyrate concentration in water fraction of the ethyl acetate extraction of acidic EcCFS (none pH-adjusted EcCFS; pH 4.5). Interestingly, the water fraction of acidic EcCFS had less butyrate concentration than that of neutralized EcCFS, which affected anti-CRC activity, suggesting that butyrate may be a key active agent of EcCFS (Fig. S3C). Furthermore, we examined the genes related to butyrate biosynthesis using whole-genome sequence data of *E. callanderi* KGMB02377. We confirmed that KGMB02377 possessed genes involved in butyrate biosynthesis (Fig. 5A). Furthermore, we assumed that the bioactive molecule of EcCFS could be other molecules; thus, we investigated the EcCFS aqueous phase of EtOAc extraction by GC-MS (Fig. 4). Five peaks between EcCFS and the RCM control were different in the chromatograms and identified by MS (Table 2). One of these five candidates, 4-aminobutanoic acid, known as γ-aminobutyric acid (GABA), has been reported to inhibit the proliferation of colon cancer cells (46). Additionally, GABA-producing Lactobacillus plantarum can induce apoptosis in 5-fluorouracil-resistant CRC cells and inhibit metastasis (47). However, the concentration of the GABA in the water fraction of EcCFS is 14.78 mM, which is lower than the concentration of GABA that exhibited antiproliferative activity (Fig. S3B). Nevertheless, we assumed that GABA might be able to show synergistic activity with butyrate; thus, we checked the antiproliferative activity of GABA with low activity concentration of butyrate. Interestingly, while butyrate and GABA showed low and no activity, respectively, the simultaneous treatment of both butyrate and GABA revealed synergistic activity (Fig. S3D), suggesting that GABA is also a possible candidate of the active agent of EcCFS. Furthermore, we analyzed whether *E. callanderi* KGMB02377 possesses GABA biosynthesis pathway genes through WGS. WGS data revealed that *E. callanderi* has several genes involved in GABA biosynthesis (Fig. 5B). In addition, other GMs that had antiproliferative activity from our previous study produce butyrate and GABA (Table S3). Although our biochemical and genomic studies suggested that the putative active molecules for antiproliferative activity in EcCFS are butyrate and GABA, further studies using inhibitors or mutants for butyrate and GABA need to be carried out. Moreover, molecules other than butyrate and GABA cannot be excluded. Indeed, in the WGS data, we discovered enzymes that are related to the biosynthesis of phytoene and lycopene (data not shown), which exert anticancer activity by inducing apoptosis and inhibiting the cell cycle (48, 49). Accordingly, future studies need to be performed to conclusively identify the active molecules involved in the antiproliferative activity of *E. callanderi*.

In this study, we confirmed the anti-CRC activity of *E. callanderi in vivo*; CT26 murine CRC cells were injected in the mice administered *E. callanderi* live bacteria and its CFS. Live bacteria and EcCFS successfully inhibited tumor growth *in vivo* (Fig. 6). Interestingly, unlike our previous study that revealed only the CFS of *O. splanchnicus*, not live bacteria, exhibits anti-CRC activity *in vivo* (14), oral administration of *E. callanderi* live bacteria inhibited tumor growth in the mouse model. We speculated that orally administered *E. callanderi* can safely reach the gastrointestinal tract, leading to successful colonization, allowing it to exert anti-CRC effects by producing the same active molecules suggested in *in vitro* studies or different molecules *in vivo*, which has not been investigated in this study. Indeed, while a previous report indicated that *O. splanchnicus* demonstrated low adhesion to epithelial cells (50), many metagenome studies have shown that *Eubacterium* is a major genus of GM across locations and races, suggesting that *Eubacterium* spp. have the ability to colonize the host gut (44). Additionally, we observed different results between *in vitro* and *in vivo* studies. We observed the anti-inflammatory activity of *E. callanderi in vivo*, indicating that live bacteria and EcCFS successfully reduced serum IL-6 levels (Fig. 7A and B), which was not observed in the *in vitro* screening using RAW 264.7 cells. Additionally, while we observed apoptosis *in vivo* and *in vitro* (Fig. 7C), we did not observe cell cycle arrest *in vivo* (data not shown). We assumed that the different results may be due to the different experimental conditions, since the tumor and colon are more complex environments than the single cell lines.

In conclusion, our study is the first to reveal that *E. callanderi* possesses anti-CRC activity. EcCFS selectively inhibited the proliferation of CRC cells and suppressed tumor progression in an allograft murine CRC model. Additionally, we speculated that the anticancer agents of *E. callanderi* may be the metabolites butyrate and GABA. Accordingly, our study suggests that *E. callanderi* could be a novel therapeutic GM for the prevention and treatment of CRC.

## MATERIALS AND METHODS

### Cell cultures.

The HCT116 and RAW 264.7 were obtained from the Korean Collection for Type Cultures (KCTC; Jeongeup, Republic of Korea). The CT26, CCD 841 CoN, and 3T3-L1 were purchased from the American Type Culture Collection (ATCC; cat. numbers CRL-2638, CRL-1790, and CL-173). All cell lines were maintained in Dulbecco’s modified Eagle’s medium (DMEM; Gibco, NY, USA) supplemented with 10% fetal bovine serum (FBS; Gibco, NY, USA), 200 U/mL of penicillin, and 100 μg/mL of streptomycin and cultured at 37°C in an incubator containing 5% CO_2_.

### Bacterial culture and preparation of EcCFS.

*E. callanderi* KGMB02377 has been isolated from the feces of a healthy Korean individual as described in our previous study (14). The identification of the strain was verified by 16S rRNA gene sequencing and phylogenetic and phenotypic analyses. Bacterial isolation and cultivation were performed in an anaerobic chamber (Coy Laboratory Products, MI, USA) that was filled with 86% N_2_, 7% CO_2_, and 7% H_2_. *E. callanderi* KGMB02377 was subcultured every 2 days on tryptic soy agar (BD, NJ, USA) supplemented with 5% sheep blood (TSAB). Bacteria were preserved at −80°C in 10% (vol/vol) skim milk solution. EcCFS was prepared as previously described (14). Briefly, *E. callanderi* KGMB02377 was inoculated into 20 mL of reinforced clostridial medium (RCM; MB cell, Seoul, Republic of Korea) broth and incubated under anaerobic conditions for 48 h. The cells were removed by centrifugation at 6,000 × *g* for 30 min, and the supernatant was harvested. To remove the effect of acidic pH, pH was adjusted using 1 M NaOH to 6.8, and filtered through a 0.22 μm pore size hydrophilic polyethersulfone membrane filter. The prepared EcCFS was stored at −20°C until use.

### Cell viability test using an MTT assay.

The MTT assay was used to determine the antiproliferative effect of EcCFS on HCT116, CT26, and CCD 841 CoN. The cells were seeded in 96-well plates and incubated for 24 h before treatment. For the treatments, the medium was replaced with fresh medium containing 10% (vol/vol) of EcCFS or RCM as control, followed by incubation in a CO_2_ incubator at 37°C. After 72 h, the medium was removed, and fresh medium containing 10% MTT solution (5 mg/mL) was added to each well and incubated for 4 h. The colored formazan crystals were solubilized using 0.04 M HCl isopropanol. Cell viability was measured at 595 nm using a microplate reader (Multiskan FC; Thermo Fisher Scientific, MA, USA). Antiproliferative activity was calculated as follows:

$$\mathrm{Anti}\mathrm{proliferative activity}\left(\text{\%}\right)=100-A_{\mathrm{treatment}}/A_{\mathrm{control}}\times 100.$$

### Flow cytometry for apoptosis and cell cycle analysis.

Apoptosis was evaluated by FITC-conjugated annexin V and PI flow cytometry using a commercial kit (Thermo Fisher Scientific, MA, USA), as previously described, with minor modifications (14). Briefly, HCT116 cells were seeded at 3 × 10^5^ cell/well in a 6-well plate and stabilized overnight. The following day, EcCFS (10% [vol/vol]) was added to the culture medium for 72 h. After 72 h of incubation, the cells were collected by trypsinization, washed twice with Dulbecco’s PBS (DPBS), resuspended in Annexin binding buffer, and labeled with FITC-conjugated Annexin V for 10 min at room temperature. After incubation, cells were resuspended in binding buffer. Finally, the cells were stained with PI solution, and the labeled cells were analyzed using an Attune NxT Flow Cytometer (Thermo Fisher Scientific, MA, USA).

For cell cycle analysis, the same method as that for apoptosis using the cell-harvesting step was applied. Harvested cells were fixed in 70% EtOH for 1 h. After fixation, the cells were washed with cold DPBS and followed by PI staining for 30 min. The cells were then analyzed using a flow cytometer. Cells treated with RCM were used as negative controls for both apoptosis and cell cycle analyses.

### Western blot analysis.

Total protein was extracted from cell lines and mouse tumors using RIPA buffer (Thermo Fisher Scientific, MA, USA), and protein concentration was measured using bicinchoninic acid (BCA) assay. Total protein (50 μg) was separated by 13% SDS polyacrylamide gel electrophoresis and transferred to a nitrocellulose membrane. The membranes were then blocked with 5% skim milk in Tris buffered saline with 0.1% Tween 20 (TTBS) and incubated with primary antibodies (1:1,000 dilution) including antibodies for cleaved-caspase3, cleaved-PARP, cyclinB1, cdc2, and β-actin (Cell Signaling, MA, USA) at 4°C overnight. After washing thrice with TTBS, the membranes were incubated with an HRP-conjugated secondary antibody (Cell Signaling Technology). Finally, the membranes were treated with ECL substrate (Thermo Fisher Scientific, MA, USA), and detection was conducted using a chemiluminescence detector (fusion solo S; Vilber). β-actin was used as loading control.

### Characterization of the EcCFS bioactive molecules.

To examine the properties of the antiproliferative agents produced by *E. callanderi*, the following three treatments were performed. First, EcCFS and RCM (as a control) were boiled at 100°C for 10 min to check their heat stability. Second, the proteinaceous nature was confirmed by treatment with proteolytic enzymes (10 μL/mL of proteinase K, trypsin, papain, chymotrypsin, and pepsin; Sigma) for 1 h at 37°C. Third, to fractionate EcCFS into an organic phase and aqueous phase, 500 mL of EcCFS or RCM was mixed and vigorously stirred with the same volume of EtOAc for 30 min at room temperature, followed by separation into two phases in a separatory funnel. The organic phase was evaporated using a rotary vacuum evaporator (Eyela, NY, USA) and dissolved in MeOH (1 mL). The aqueous phase was briefly evaporated using a rotary vacuum evaporator to remove trace amounts of EtOAc from aqueous phase.

Short-chain fatty acids in EcCFS were measured using an HPLC system (Shimadzu, Tokyo, Japan). Normal-phase analytical HPLC experiments were performed using Aminex Organic Acid Columns (Bio-Rad, CA, USA). The isocratic method was used with 5 mM sulfuric acid (H_2_SO_4_) as the mobile phase at a flowrate of 0.7 mL/min for 25 min. The obtained peaks were analyzed by comparison with the respective SCFA standard peaks. For the quantification of SCFA, SCFAs mix was measured as standard at a wavelength of 210 nm and the calibration curve of each SCFA was drawn to calculate the amount of SCFA in EcCFS.

For GC-MS analysis, the dried aqueous phase of EcCFS was derivatized with 20 mg/mL methoxamine hydrochloride (Sigma, MO, USA) solution in pyridine and incubated at 30°C for 90 min with shaking. Subsequently, the extracts were trimethylsilylated for 30 min at 37°C with N-methyl-N-trimethylsilyl trifluoroacetamide (MSTFA; Sigma, MO, USA). The derivatized extracts were analyzed using an Agilent 7890 B gas chromatograph coupled with an Agilent 7000C mass-selective detector (Agilent Technologies, CA, USA). The 1-μL aliquots of the extracts were injected into a VF-5ms column (30 m × 250 μm inner diameter [I.D.], 0.5 μm film thickness; Agilent Technologies, CA, USA) using an injector tower G4513A (Agilent Technologies, CA, USA) in split less mode. The initial GC oven temperature was 70°C; 5 min after injection, the GC oven temperature was increased at a rate of 5°C/min to 320°C and held there for 5 min. Helium was used as the carrier gas, and the helium flow was kept constant at a flow rate of 1.7 mL/min. Detection was achieved using MS in electron impact mode and full scan monitoring mode (*m/z* 15 to 800).

### Genome analysis.

WGS was performed using the Illumina NovaSeq technology (Illumina, CA, USA) at CJ Bioscience. The paired-end reads obtained were assembled using SPAdes (version 3.13.0) after quality trimming. Completeness and contamination of the assembled genome were examined using ContEst16S and CheckM tools. The coding DNA sequences (CDSs) and tRNA were predicted using Prodigal and tRNAscan-SE, respectively. The rRNAs were identified using a covariance model search with inference of Rfam 12.0. The annotation of each CDS was performed by a homology search against the Swiss-Prot, EggNOG 4.5, SEED, and KEGG databases. Butyrate and GABA synthesis-related genes in *E. callanderi* KGMB02377 were analyzed in the sequenced genome by a BLAST search with reference genes (25, 51).

### Animal experiments.

A total of 32 male BALB/c mice (6 to 8 weeks old) were obtained from Kosa Bio, Inc. (Seongnam, Republic of Korea). Mice were maintained at 22 ± 2°C, 55% ± 5% humidity, and 12 h light/dark cycle under specific pathogen-free conditions. Commercial rodent diet and water were supplied freely to the mice. All mouse experiments were approved by the Institutional Animal Care and Use Committee of the Korea Research Institute of Bioscience and Biotechnology (approval number KRIBB-AEC-20095), and all animals were cared for according to the guidelines for animal experiments of the KRIBB. For the CRC murine model, CT26 cells were suspended in DPBS at a concentration of 4 × 10^7^ cells/mL, and 50 μL was injected subcutaneously into the flank of each mouse. Tumor size (V) was measured three times per week via the length and width of the tumor using the following formula:

$$\text{Tumor size}(\text{V})=\mathrm{widt}h^{2}\times \mathrm{length}\times 0.5$$

With regard to *E. callanderi* live bacteria treatment, mice were pretreated with bacteria by oral administration of 1 × 10^8^ CFU/100 μL/mouse/day for 2 weeks prior to tumor injection, and bacteria were administered weekly after tumor injection. For EcCFS treatment, 200 μL of EcCFS was peri-tumorally injected twice per week around the tumor, 4 days after the initial inoculation of cancer cells. PBS and RCM were used as negative controls for live bacteria and EcCFS treatment, respectively.

### ELISA.

IL-6 ELISA analysis was conducted according to the manufacturer’s instructions (BD Biosciences, NJ, USA). Briefly, the IL-6 capture antibody was attached to a microplate overnight at 4°C. The IL-6 standards and diluted mouse serum (2-fold dilution) were added and incubated to bind to the capture antibody. After 24 h of incubation, the detection antibody was added and the cells were incubated for 1 h. The SAV-HRD reagent was added for 1 h. The HRP substrate was added, and the absorbance was measured at 450 nm using a spectrophotometer.

### Statistical analysis.

All statistical analyses were performed using GraphPad Prism (USA). Data are presented as mean ± standard deviation (SD) or as mean ± standard error of mean (SEM). Statistical significance was calculated with Student's *t* test. Significance was accepted at the level of *, *P < *0.05; **, *P < *0.01.

### Data availability.

The GenBank/EMBL/DDBJ accession number for the whole-genome sequence of strain KGMB02377 is JAHMUG000000000.
