# Supplementary material for: Gut Microbiota Eubacterium callanderi Exerts Anti-Colorectal Cancer Activity
Source: Microbiol Spectr. 2022 Nov 30;10(6):e02531-22. doi: 10.1128/spectrum.02531-22 (PMC9769849; doi:10.1128/spectrum.02531-22)
Supplement: Supplemental file 1 — Supplemental material. Download spectrum.02531-22-s0001.pdf, PDF file, 2.4 MB [file spectrum.02531-22-s0001.pdf]

Microbiology spectrum

Gut Microbiota *Eubacterium callanderi* Exerts Anti-colorectal Cancer Activity

Seoung Woo Ryu[a†], Ji-Sun Kim[a†], Byeong Seob Oh[a†], Won Jung Choi[a], Seung Yeob Yu[a], Jeong Eun Bak[a], Seung-Hwan Park[a], Se Won Kang[a], Jiyoung Lee[a], Won Yong Jung[b], Jung-Sook Lee[a], Ju Huck Lee[a]

[a] Korean Collection for Type Cultures, Biological Resource Center, Korea Research Institute of Bioscience and Biotechnology, Jeongeup 56212, Republic of Korea

[b]Korean Bioinformation Center, Korea Research Institute of Bioscience and Biotechnology, Daejeon 34141, Republic of Korea.

† These authors have contributed equally to this work and share first authorship.

Correspondences:

Jung-Sook Lee, jslee@kribb.re.kr, Ju Huck Lee, juhuck@kribb.re.kr

Running Head: Anti-colorectal Cancer Activity of *E. callanderi*

Supplementary Materials

Supplementary Figures

Figure S1. Identification of *E. callanderi* KGMB02377. **(A)** Phylogenetic tree showing the position of *E. callanderi* KGMB02377 among the core species of the genus *Eubacterium* based on 16S rRNA sequences by the neighbor-joining, where the nucleotide substitution model of Kimura-2 parameter was employed. Numbers at nodes refer to bootstrap values (based on 1000 replicates, only values >50% are shown at branch points). Filled circles indicate that the corresponding nodes (groupings) were recovered by the neighbor-joining and maximum-likelihood methods. Bar, 2% sequence divergence. **(B)** Cell morphology of *E. callanderi* KGMB02377, as observed with transmission electron microscope (left) and scanning electron microscope (right).

Figure S2. *In vitro* screening of inhibitory activity of EcCFS against various diseases. **(A)** Anti-proliferation effect of EcECFS on HCT116 cells. **(B-D)** Inhibitory activity of EcCFS on inflammation parameters; NO production (B), IL-6 production (C), and TNF-α production (D) in LPS-treated RAW 264.7 cells. **(E)** *C. difficile* growth inhibition effect of EcCFS. **(F)** Inhibitory activity of EcCFS on adipogenic differentiation of 3T3-L1 cells. RCM was used as control in all assays.

Figure S3. Anti-proliferative activity of bioactive molecules **(A)** Dose-dependent anti-proliferative activity of butyrate in HCT116. **(B)** Dose-dependent anti-proliferative activity of GABA in HCT116. **(C)** pH effect on anti-proliferative activity of EcCFS aqueous phase. The butyrate concentration of EcCFS aqueous phase in pH 4.5 and pH 6.8 were 4.56 mM and 10.2 mM, respectively. **(D)** Synergistic anti-proliferation effects of butyrate and GABA in HCT116. 0.3 mM butyrate and 100 mM GABA were treated.

Figure S4. Schematic diagram showing the experimental design of the colorectal cancer murine models. **(A)** Schematic diagram of the orally administrated CRC murine model; PBS or *E. callanderi* were orally administrated ($1x10^8$ CFU/100μl/mouse/day) for 2 weeks prior to tumor injection. **(B)** Schematic diagram of peri-tumoral CRC murine model. Filled arrows indicate oral administration or peri-tumoral injection schedules, and empty arrow indicates the subcutaneous injection of CT26 ($2x10^5$ cells/50μl).

Supplementary Tables

Table S1. The genome statistics of *E. callanderi* KGMB02377

**Properties**	**Values**
Genome assembly	
Assemble method	SPAdes 3.13.0
Genome coverage	385.6 X
Genome features	
Genome size (bp)	4,673,496
G+C content (mol%)	47.2
No. of contigs	43
rRNA genes (5S, 16S, 23S)	5 (2, 1, 2)
tRNA genes	43
Open reading frame	4,429
CDS assigned by COG	3,799
GenBank Accession No.	JAHMUG000000000

Table S2. Clusters of orthologous groups (COG) of proteins of *E. callanderi* KGMB02377

COG	Description	Number of Genes	%
**J**	Translation, ribosomal structure and biogenesis	154	4.1%
**K**	Transcription	363	9.6%
**L**	Replication, recombination and repair	176	4.6%
**D**	Cell cycle control, cell division, chromosome partitioning	27	0.7%
**O**	Posttranslational modification, protein turnover, chaperones	79	2.1%
**M**	Cell wall/membrane/envelope biogenesis	174	4.6%
**N**	Cell motility	8	0.2%
**P**	Inorganic ion transport and metabolism	193	5.1%
**T**	Signal transduction mechanisms	226	5.9%
**C**	Energy production and conversion	242	6.4%
**G**	Carbohydrate transport and metabolism	218	5.7%
**E**	Amino acid transport and metabolism	299	7.9%
**F**	Nucleotide transport and metabolism	78	2.1%
**H**	Coenzyme transport and metabolism	141	3.7%
**I**	Lipid transport and metabolism	54	1.4%
**Q**	Secondary metabolites biosynthesis, transport and catabolism	33	0.9%
**R**	General function prediction only	0	0.0%
**S**	Function unknown	1334	35.0%
**Total**		3799	100%

Table S3. Concentration of butyrate and GABA in CFS of gut microbiota with anti-proliferative activity.

**Butyrate and GABA -producing GM**	**Butyrate (mM)**	**GABA (mM)**	**Anti-proliferative effect on HCT116 (%)**
***E. callanderi* KGMB02377**	**10.20**	**14.78**	**54.42**
***Bacteroides* spp. 1**	**0.90**	**6.72**	**29.77**
***Bacteroides* spp. 2**	**0.44**	**12.49**	**26.39**
***Bacteroides* spp. 3**	**1.32**	**2.23**	**29.49**
***Bacteroides* spp. 4**	**0.90**	**13.53**	**32.84**
***Bacteroides* spp. 5**	**0.46**	**6.34**	**27.21**
***Parabacteroides* spp. 1**	**0.53**	**11.33**	**33.38**
***Parabacteroides* spp. 2**	**0.54**	**4.93**	**27.57**

A

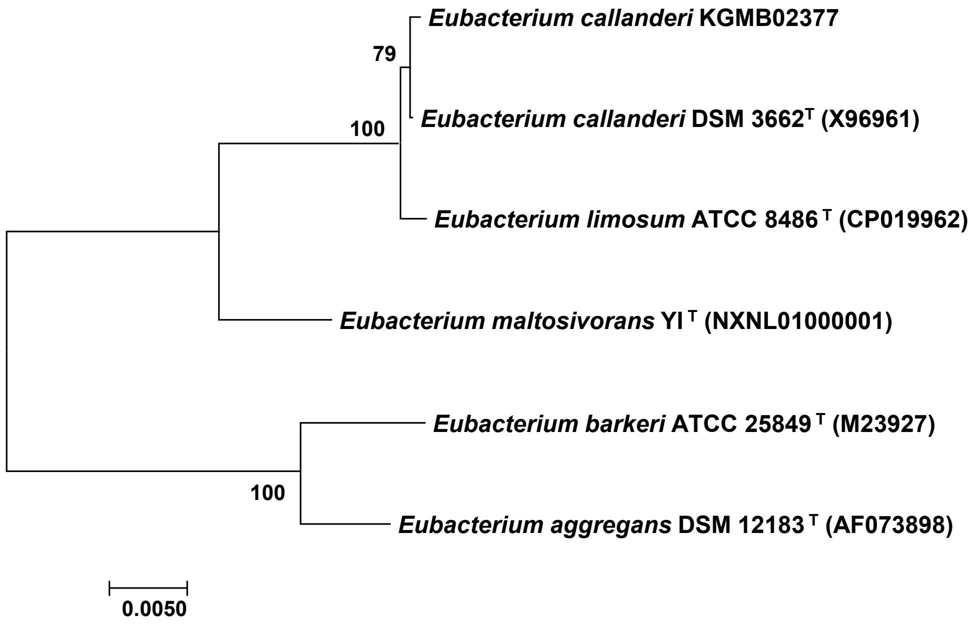

Eubacterium callanderi KGMB02377
79
Eubacterium callanderi DSM 3662T (X96961)
100
Eubacterium limosum ATCC 8486 T (CP019962)
Eubacterium maltosivorans YI T (NXNL01000001)
Eubacterium barkeri ATCC 25849 T (M23927)
100
Eubacterium aggregans DSM 12183 T (AF073898)
0.0050

B

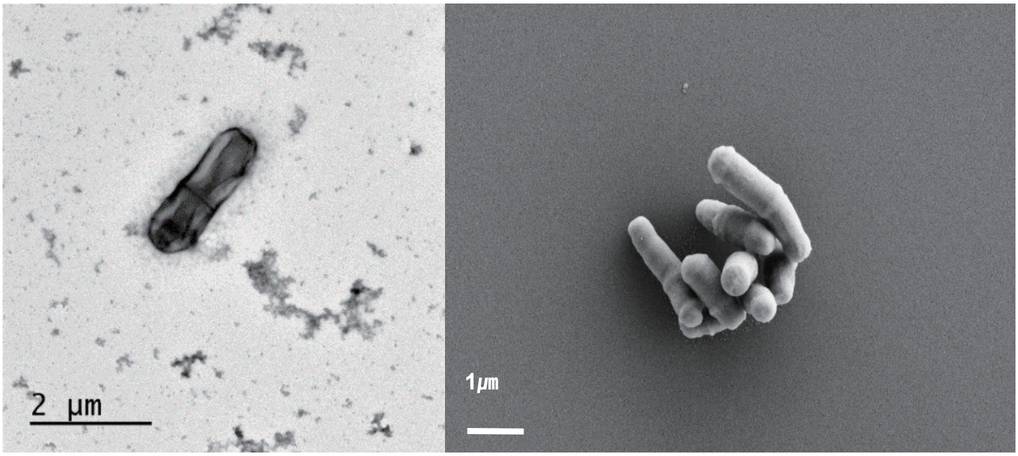

2 μm
1μm

Fig S1.

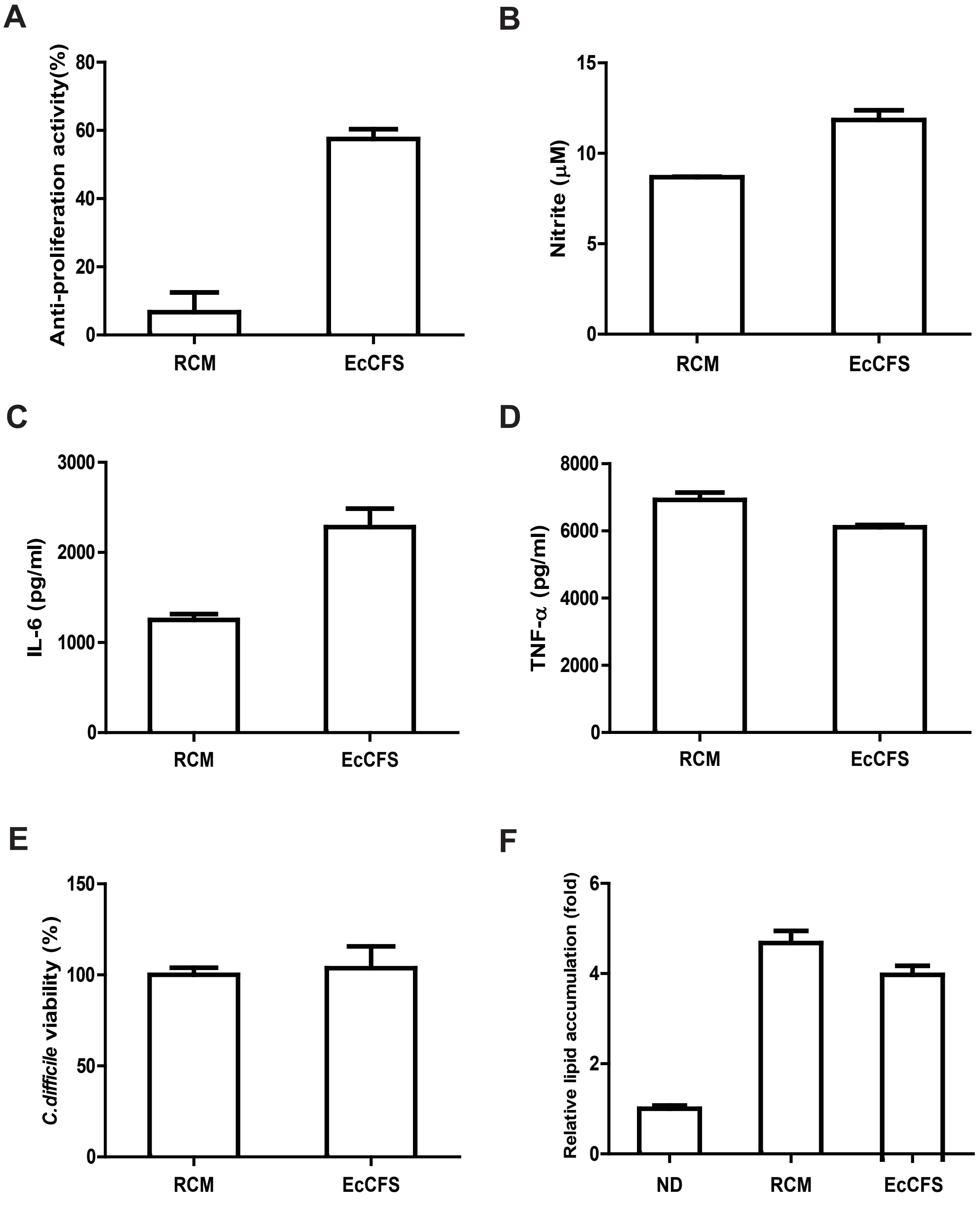

A
Anti-proliferation activity(%)
0
20
40
60
80
RCM
EcCFS
B
Nitrite (μM)
0
5
10
15
RCM
EcCFS
C
IL-6 (pg/ml)
0
1000
2000
3000
RCM
EcCFS
D
TNF-α (pg/ml)
0
2000
4000
6000
8000
RCM
EcCFS
E
C.difficile viability (%)
0
50
100
150
RCM
EcCFS
F
Relative lipid accumulation (fold)
0
2
4
6
ND
RCM
EcCFS

Fig S2.

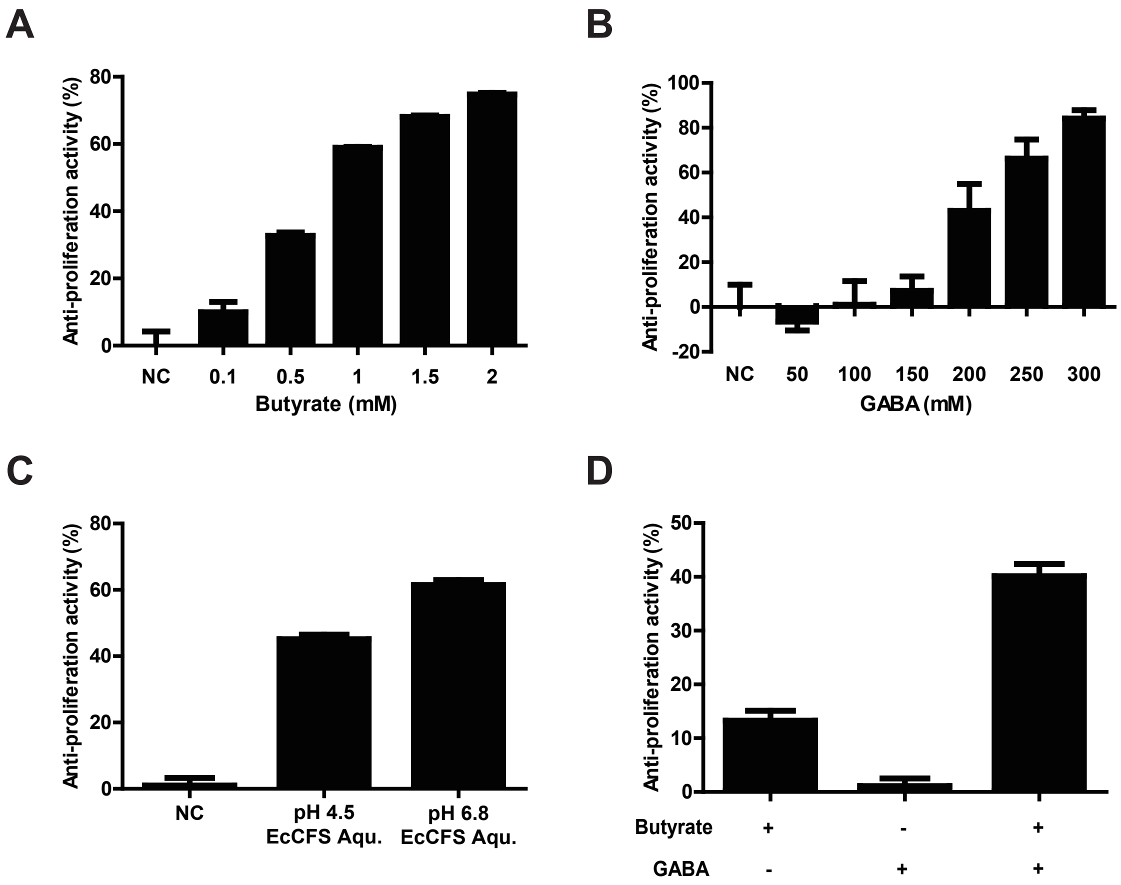
A
Anti-proliferation activity (%)
80
60
40
20
0
NC
0.1
0.5
1
1.5
2
Butyrate (mM)
B
Anti-proliferation activity (%)
100
80
60
40
20
0
-20
NC
50
100
150
200
250
300
GABA (mM)
C
Anti-proliferation activity (%)
80
60
40
20
0
NC
pH 4.5 EcCFS Aqu.
pH 6.8 EcCFS Aqu.
D
Anti-proliferation activity (%)
50
40
30
20
10
0
Butyrate + - +
GABA - + +

Fig. S3

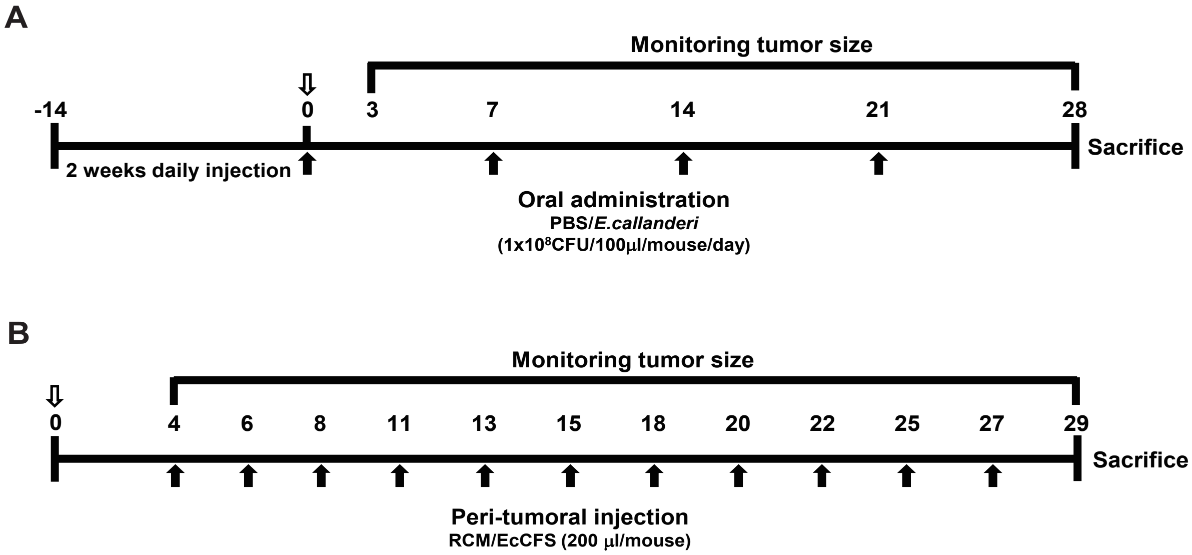
A
Monitoring tumor size
-14
0
3
7
14
21
28
Sacrifice
2 weeks daily injection
Oral administration
PBS/E.callanderi
(1x10^8CFU/100μl/mouse/day)
B
Monitoring tumor size
0
4
6
8
11
13
15
18
20
22
25
27
29
Sacrifice
Peri-tumoral injection
RCM/EcCFS (200 μl/mouse)

Fig S4.
